# Supplementary material for: Communicating Personal Melanoma Polygenic Risk Information: Participants’ Experiences of Genetic Counseling in a Community-Based Study
Source: J Pers Med. 2022 Sep 26;12(10):1581. doi: 10.3390/jpm12101581 (PMC9605561; doi:10.3390/jpm12101581)
Supplement: Supplementary file 1 [file jpm-12-01581-s001.zip › jpm-1915513-supplementary.pdf]

The Managing Your Risk Study Group: Louise Keogh, Ainsley Newson, Graham Mann, Hugh Dawkins, Jacqueline Savard, Lyndal Trevena, Phyllis Butow, Brooke Beswick, Gillian Reyes-Marcelino, Cynthia Low, Ashleigh Sharman, Rachael Morton, Michael Kimlin, Kate Dunlop, Matthew H. Law, Martin Allen, Serigne Lo, Peter A Kanetsky.

Supplementary Table S1. Assessment of fidelity to the genetic counsellor telephone manual

Topic	Proportion reached adequate fidelity (%) N=17
Confirmation that booklets were received	100
Result recall and explanation	100
Explanation of melanoma risk factors	100
Gauge participant feelings about results	100
Explanation of prevention and early detection behaviours	94
Offer the chance to ask more questions about results	75
Outline next part of research study	88
Offer the chance to ask any additional questions	75
